# Supplementary material for: Learning curve and surgical outcome of robotic assisted colorectal surgery with ERAS program
Source: Sci Rep. 2022 Nov 29;12:20566. doi: 10.1038/s41598-022-24665-w (PMC9709162; doi:10.1038/s41598-022-24665-w)
Supplement: Supplementary file 1 — Supplementary Information 1. [file 41598_2022_24665_MOESM1_ESM.zip › LSC Raw data-Ñ[▒K/119/N_ñw╝╨░O▒Kñσ.pdf]

W52 / W52

# 臺中榮民總醫院
## 手術室護理記錄

[redacted]

## 基本資料

- 室別： 二醫手術室-31室，手術科別： CRS，手術類別： 常規，日期： 2021/03/04
- 進入時間： 1310 ； 麻醉開始時間： 1310 ； Time Out時間： 1435 ； 劃刀時間： 1437 ； 手術結束時間： 1825 送出時間： 1830 ；
- 麻醉方式： GE
  診斷：Malignant tumor of A-colon
  手術名稱：Laparoscopic right colectomy and anastomosis
  手術局部用藥：nil
  術前預防性抗生素：
  - 抗生素名稱一： Veterin 1GM IVD/由麻醉護理師給予
  - 時間： 1425
  - 上次簽章: 朱詠暄
  
  ，追加預防性抗生素：
- 手術醫師： 蔣鋒帆，黃俋霖，楊曙榮，翁明謙
- 麻醉醫師： VS 張思玲
-
  - 刷手護士A ： 李庭，時間： 1310-1800，接替刷手一： 陳昱穎，時間： 1800
  - 流動護士A ： 朱詠暄，時間： 1310，接替流動一： 陳昱穎，時間： 1500-1730，接替流動二： 葉新娟，時間： 1730

---

## 皮膚狀況

A-V Shunt： 無
術前： 完整
記錄者： 上次簽章: 朱詠暄
術後： 完整
記錄者： 上次簽章: 陳昱穎

---

## 手術準備

手術臥式 單一手術臥式

-
  - 手術臥式： 仰臥
  - 使用附件： 溫毯 38 ℃，頭圈
  - 皮膚準備： 10%Aq Betadine，Hibiscrub，2% Chlorhexidine
  - 電燒： 使用電燒: 電極片位置： 其它： 電燒墊，記錄者： 上次簽章: 朱詠暄

止血帶T： 無

---

## 植入物

- 植入物名稱/號碼/數量/部位：surgiwrap/小/壹/abdominal

，記錄者： 上次簽章: 陳昱穎

---

## 標本送檢

- 細菌培養 無
- 細胞學 無
- 冰凍切片 無
- 病理標本
  組織名稱一：A COLON
- 截肢 無

標本交付 標本： 1 袋，標本： 零 盒，標本： 零 瓶，細胞學： 零 管，Culture Tube： 零 管，被交付醫師： CR 楊曙榮，

- 記錄者： 上次簽章: 葉新娟
- 時間： 1830

## 傷口術後敷料

傷口敷料： 傷口敷料： 無

- 請於圖上標示部位：

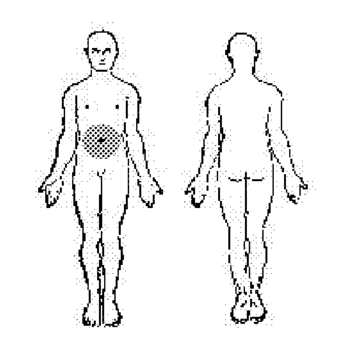

腹部肆切口.正中直切

- 傷口敷料： MENPORE

## 導管

| 導管 | 天數 | 導管資訊 | 插入日期 | 插入時間 | 更換日期 | 拔除日期 | 拔除時間 | 拔除原因 | 預計拔除/更換 | 預計拔除/更換日期 |
|---|---|---|---|---|---|---|---|---|---|---|
| A-line (1) | 1 | 號碼:22 號; 部位:左手; | 2021/03/04 | 1410 | | | | | | |
| Foley(1) | 1 | 號碼:16 Fr.; 部位:膀胱; | 2021/03/04 | 1400 | | | | | | |

## 用物計數

- 手術中出血量： 微 c.c.
- 手術中尿量： 500 c.c.
- 
  - 其它： 無

用物計數： 單一切口

- 1.器械： 正確，2.縫針： 正確，3.刀片： 正確，4.紗布： 正確，5.腹部墊： 未使用，6.紗球： 未使用，7.PEANUTS ： 未使用，8.紗條： 未使用，9.絲帶： 未使用，10.針頭： 未使用，11.Vessel loop： 未使用，12.Boots： 未使用，13.Jelco： 未使用，14.棉條： 未使用，
  - 第一次：
    - 刷手護士 ： 李庭
    - 流動護士： 上次簽章: 葉新娟
    - 時間： 1730

  ,
  - 第二次：
    - 刷手護士 ： 李庭
    - 流動護士： 上次簽章: 葉新娟
    - 時間： 1750

  ,
  - 第三次：
    - 刷手護士 ： 陳昱穎
    - 流動護士： 上次簽章: 葉新娟
    - 時間： 1800

## 備註

備註：NIL

- 記錄者： 上次簽章: 朱詠暄
- 時間： 1500

填寫護理人員 葉新娟

正式摘要

W52 / W52

doctorRank:B
